# Identification immune-related hub genes in diagnosing atherosclerosis with ischemic stroke through comprehensive bioinformatics analysis and machine learning

**DOI:** 10.3389/fneur.2025.1507855

**Published:** 2025-04-30

**Authors:** Ming Zhang, Li-Jun Tang, Shi-Yu Long

**Affiliations:** ^1^Yilong County People's Hospital of Nanchong, Nanchong, China; ^2^Department of Neurology, Gaoping District People's Hospital of Nanchong, Nanchong, China

**Keywords:** ischemic stroke, atherosclerosis, WGCNA, machine learning, immune cell infiltration, bioinformatics

## Abstract

**Background:**

Atheroma plaques are major etiological factors in the pathogenesis of ischemic stroke (IS). Emerging evidence highlights the critical involvement of the immune microenvironment and dysregulated inflammatory responses throughout IS progression. Consequently, therapeutic strategies targeting specific immune-related markers or signaling pathways within this microenvironment hold significant promise for IS management.

**Methods:**

We integrated Weighted Gene Co-expression Network Analysis (WGCNA), CIBERSORT, and machine learning (LASSO/Random Forest) to identify disease-associated modules and hub genes. Immune infiltration analysis evaluated hub gene-immune cell correlations, while protein-protein interaction (PPI) and ROC curve analyses assessed diagnostic performance.

**Results:**

Comprehensive bioinformatics analysis identified three hub genes—OAS2, TMEM106A, and ABCB1—with high prognostic value for ischemic stroke. Immune infiltration profiling revealed significant correlations between these genes and distinct immune cell populations, underscoring their roles in modulating the immune microenvironment. The diagnostic performance of the gene panel was robust, achieving an area under the curve (AUC) was calculated as 0.9404 (*p* < 0.0001; 95% CI: 0.887–0.9939) for atherosclerotic plaques, demonstrating superior accuracy compared to conventional biomarkers.

**Conclusion:**

By integrating machine learning with multi-omics bioinformatics, we established a novel three-gene signature (OAS2, TMEM106A, ABCB1) for precise diagnosis of atherosclerosis and ischemic stroke. These genes exhibit dual diagnostic utility and may influence disease progression through immune cell modulation. Our findings provide a foundation for developing targeted therapies and biomarker-driven clinical tools.

## Introduction

Stroke is a serious neurological disorder caused by an interruption or reduction in blood supply to the brain, leading to brain cell death and various neurological deficits (1). It primarily occurs in two forms: ischemic stroke, which results from a blockage in arteries supplying the brain, and hemorrhagic stroke, caused by bleeding into or around the brain (2).

Ischemic stroke (IS) can be further classified into several subtypes based on the underlying cause of the blockage, including large artery atherosclerosis, cardioembolism, small-vessel occlusion, stroke of other determined cause, and stroke of undetermined cause (3). Among these, atherosclerosis is a common cause of ischemic stroke.

Atherosclerosis, a chronic inflammatory disorder, initiates with endothelial dysfunction and low-density lipoprotein (LDL) retention in arterial walls, triggering myeloid cell infiltration and foam cell formation (4, 5). This cascade culminates in plaque development, where immune-mediated processes dictate stability: vulnerable plaques rich in inflammatory macrophages and neutrophils predispose to rupture, whereas stable plaques exhibit fibrous caps and regulatory T-cell (Treg) enrichment (6–8). Over time, these plaques can harden and narrow the arteries, reducing blood flow. Atheroma plaques are a significant contributor to ischemic stroke, and their transcriptomic analysis can provide valuable insights into the of IS.

The immune microenvironment orchestrates both atherosclerosis progression and post-ischemic neuronal injury (9, 10). Pro-inflammatory cytokines, neutrophil extracellular traps (NETs), and microglial activation exacerbate blood-brain barrier disruption and infarct expansion (5, 11, 12).

Despite advances, current diagnostic modalities—including ultrasonography, CT angiography, and lipid profiling—lack sensitivity for early atherosclerosis detection and fail to predict plaque vulnerability (1, 13, 14). Inflammatory biomarkers (e.g., CRP) exhibit limited specificity, highlighting the unmet need for mechanistically grounded diagnostic tools (15).

High-throughput transcriptomics has revolutionized disease mechanism exploration, enabling systematic identification of stroke-associated genes (16, 17). Here, we integrated Weighted Gene Co-expression Network Analysis (WGCNA)—a systems biology approach to detect co-expressed gene modules—with machine learning algorithms to prioritize hub genes (17–22). Unlike conventional analyses, this strategy disentangles complex interactions within atheroma microenvironments while minimizing high-dimensional data overfitting.

Our study identifies OAS2, TMEM106A, and ABCB1 as key regulators bridging atherogenesis and IS. Through protein-protein interaction (PPI) networks and ROC analysis, we validate their diagnostic efficacy for plaque instability (AUC = 0.9404) and IS risk stratification (AUC = 0.7075). These findings illuminate immune-centric pathways in stroke pathogenesis, offering translational potential for precision diagnostics and targeted therapies.

## Methods

### Ethical approval

The data used in this study are all from existing publications or public databases with ethical approval and informed consent, and no additional ethical approval is required.

### Data source

The dataset of GSE22255 and GSE43292 from GEO websites (https://www.ncbi.nlm.nih.gov/geo/). The GSE22255 was blood genomic expression profile for ischemic stroke (IS), including 20 control and 20 IS sample (23). The GSE43292 was the genome-wide expression study of human carotid atheroma, including 32 normal tissue and 32 atheroma plaque samples (24).

### Identification of the differentially expressed genes

“Limma” package (25) (version 3.56.2) was utilized to identify the DEGs between the Atheroma plaque and normal tissue. A *p* < 0.05 and |log FC (fold change)| > 0.25 were considered statistically significant. The “ggplot2” package (version 3.5.1) was used to establish a volcano plot of the DEGs. The upregulated and downregulated gene lists were sorted by logFC in each dataset.

### Weighted gene co-expression network analysis (WGCNA)

WGCNA (26) (version 1.72) is a systems biology method employed to construct gene co-expression networks, identifying clusters or modules of highly correlated genes across various samples. We applied WGCNA to analyze the top 50% of genes, exhibiting high variance in expression. Initially, a standard scale-free network was utilized to approximate the optimal soft threshold power (soft power = 18). Subsequently, adjacency values among genes, with a variance exceeding all variance quartiles, were calculated using a power function. These adjacency values were then transformed into a topological overlap matrix (TOM), from which the corresponding dissimilarity values (1-TOM) were derived. Lastly, the relationships between the modules and clinical traits were evaluated using Pearson correlation analysis, facilitating the identification of biologically significant modules.

### Construction of the LASSO model and random forest

We utilized the Least Absolute Shrinkage and Selection Operator (LASSO) logistic regression [analyzed by the “glmnet” R package (version 4.1) (22)], Random Forest (RF) as analyzed by the “randomForest” R package (version 4.7), to screen candidate genes, and the overlapping genes of the two algorithms were regarded as diagnostic markers.

### Immune cells infiltration analysis

The versatile computational method known as CIBERSORT (27) was employed to accurately quantify the fractions of immune cells within atheroma plaque and normal tissue gene expression datasets, utilizing specific immune cell signatures consisting of 3,812 differential expression genes. These genes exhibit high sensitivity and specificity for identifying 22 distinct phenotypes of human immune cells. Subsequently, samples were subjected to filtering based on a *P*-value threshold of < 0.05, following which the proportions of each immune cell type were computed. Correlations among the 22 immune cell populations were visualized using the “corrplot” package (version 0.92).

### Functional enrichment analysis

For a more comprehensive exploration of the potential biological roles of DEGs, we employed the “ClusterProfiler” package (version: 3.18.0) and the “org.Hs.eg.db” (version: 3.17.0) package within the R software environment. These tools facilitated Gene Ontology (GO) function enrichment analysis and Kyoto Encyclopedia of Genes and Genomes (KEGG) pathway enrichment analysis, with a significance threshold set at q value < 0.05. The GO analysis was instrumental in annotating gene functions, particularly focusing on biological pathways (BP), cellular components (CC), and molecular functions (MF). Meanwhile, KEGG analysis enabled the identification of enriched pathways among the DEGs.

### Correlation analysis of hub genes and the immune status

To examine the relationship between hub genes' expression levels and the immune status, we performed Spearman's rank correlation analysis using the “ggcorrplot” package (version: 0.1.4.1) in R. This analysis aimed to depict the correlations between the expression levels of hub genes and various aspects of immune status.

### ROC curve and protein-protein interaction network

The protein-protein interaction network, with genes as nodes, expresses the edge with the mapping interaction of genes encoding proteins, thus forming an undirected graph. Using the STRING database, a comprehensive dataset containing functional connections between proteins, based on experimental evidence of protein-protein interactions and interactions predicted through comparative genomics and text mining. STRING uses a scoring system designed to reflect evidence of predicted interactions. In this study, we included interactions with a score of at least 0.4, which corresponds to a medium confidence network. The diagnostic value of this key gene for atherosclerotic plaque and ischemic stroke was evaluated by receiver operating characteristic (ROC) curves. R software (version 4.3.0) and Adobe Illustrator CS6 was utilized for statistical analysis and drawing. *P*-value < 0.05 was considered statistically significant.

### Nomogram prediction model

To evaluate the predictive capabilities of hub genes related to disease occurrence and progression, we will construct a nomogram based on a logistic regression model. Logistic regression analysis will identify significant factors associated with the outcome, with each variable assigned a regression coefficient to estimate its contribution to the model. Utilizing the R package “rms” (version: 6.8), we will create the nomogram, linking each predictor to a corresponding score, which enables the calculation of a total score representing the predicted probability of the outcome.

## Results

### Study design and differential gene expression analysis

**Figure 1:**
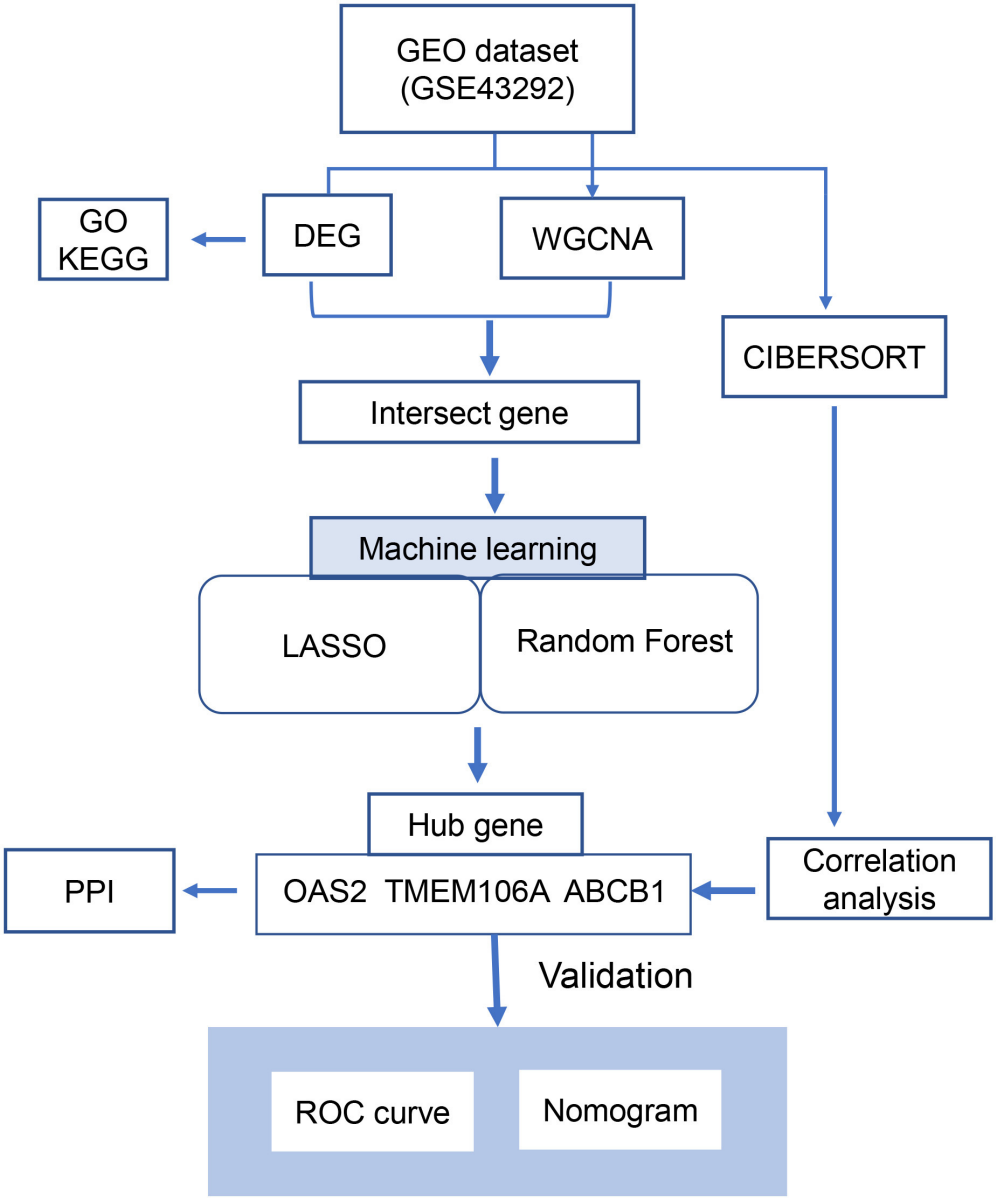
Overview of the study design.

The schematic of this study is illustrated in Figure 1. Initially, differentially expressed genes (DEGs) were identified from the GSE43292 dataset; subsequently, biologically significant modules were detected using the Weighted Gene Co-expression Network Analysis (WGCNA) method. The CIBERSORT algorithm was utilized to quantify the fractions of immune cells within atheroma plaque gene expression datasets, using specific immune cell signatures. Functionally relevant DEGs were screened by overlapping the identified DEGs with the genes in the brown module through a Venn diagram analysis. To identify the key genes with the highest prognostic significance for ischemic stroke, the Least Absolute Shrinkage and Selection Operator (LASSO) and Random Forest algorithms were applied. This approach identified three pivotal hub genes: OAS2, TEME106A, and ABCB1. The diagnostic value of these three key genes for atherosclerotic plaque and ischemic stroke was further evaluated using protein-protein interaction (PPI) network analysis, receiver operating characteristic (ROC) curve analysis, and a nomogram model.

**Figure 2:**
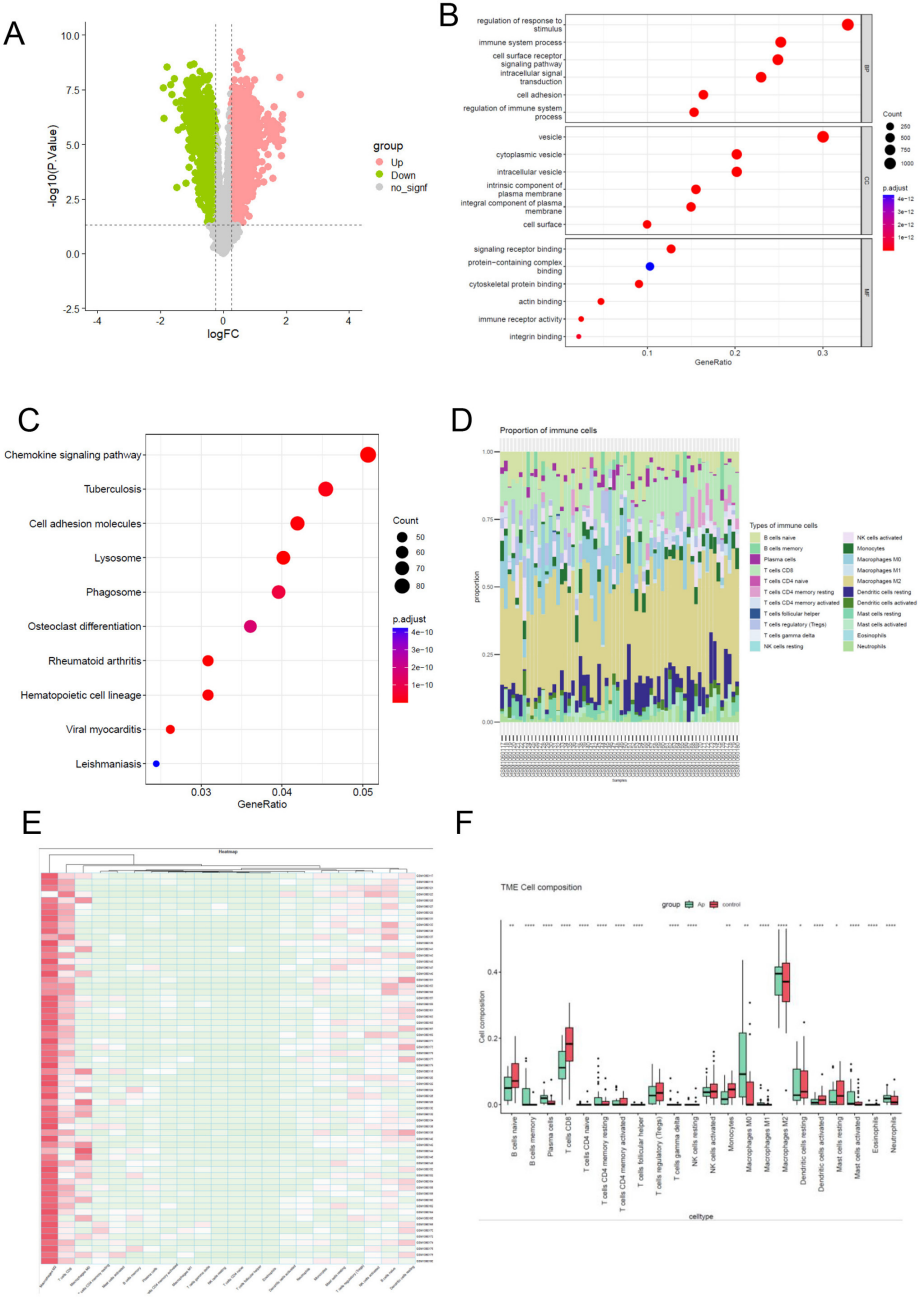
Functional enrichment analysis and the immune infiltration landscape of differential expression gene. **(A)** the volcano of differentially expressed genes between Atheroma plaque compared with normal tissue. A *p* < 0.05 and |log FC (fold change)| > 0.25 were considered statistically significant. Grenn blot represent downregulated and pink blot represent upregulated. **(B)** To analyze potential mRNA targets, using the Gene Ontology (GO) system. The ClusterProfiler utility within R software facilitated the clustering of prospective targets based on biological pathways (BP), molecular functions (MF), and cellular components (CC). A significance threshold of q value <0.05 was applied to determine statistical significance in the enrichment results. **(C)** Enriched KEGG signaling pathways were selected to illustrate the significant biological activities associated with potential mRNA. The gene ratio is represented on the abscissa, while the enriched pathways are depicted on the ordinate. **(D, E)** The proportion of 22 immune cell subpopulations in 64 samples from the GSE43292 datasets. **(F)** The disparity in immune infiltration between atheroma plaque and healthy controls was examined. The normal controls group was color-coded red, while the atheroma plaque group was color-coded green. Statistical significance was defined as a *P* < 0.05.

Differentially expressed genes were screened using the “limma” package with a threshold of *p* < 0.05 and |logFC| > 0.25). From the GSE43292 dataset, a total of 3,812 DEGs were identified, comprising 1,989 upregulated genes and 1,823 downregulated genes. The volcano plot illustrating these DEGs is presented in Figure 2A.

### Functional enrichment analysis of DEGs

To gain deeper insights into the biological mechanisms and signaling pathways associated with differentially expressed genes (DEGs) in atheroma plaque, Gene Ontology (GO) and Kyoto Encyclopedia of Genes and Genomes (KEGG) analyses were conducted. The results of GO analysis demonstrated that the DEGs were primarily enriched in processes related to the immune system, regulation of response to stimuli, cell surface receptor, cell adhesion (Figure 2B).

Similarly, the KEGG analysis revealed that the DEGs were significantly associated with pathways such as the chemokine signaling pathway, cell adhesion molecules, phagosome (Figure 2C). These findings highlight the critical involvement of immune system regulation and cell adhesion processes in the progression of atheroma plaques, providing valuable insights into the underlying molecular mechanisms and potential therapeutic targets.

### Immune infiltration analyses

To comprehensively characterize immune cell infiltration in atheroma plaque, we applied the CIBERSORT algorithm (version 1.06) with the LM22 leukocyte gene signature matrix (1,000 permutations, *p*-value threshold < 0.05 for deconvolution reliability) to RNA-seq data from GSE43292, including atheroma plaque samples and normal control tissues. Raw counts were normalized using the TMM method in edgeR to correct for library size variation, and batch effects were minimized via ComBat prior to analysis. The proportions of different infiltrating immune cell types between atheroma plaque and control tissues were shown in Figures 2D, E. Obviously, Macrophages M2 accounted for the majority of all infiltrating cells. In comparison to normal tissue, atheroma plaque exhibited higher levels of memory B cells, plasma cells, resting memory CD4 T cells, M0 macrophages, Neutrophils and activated Mast cells. Conversely, the percentages of naive B cells, CD8 T cells, activated memory CD4 T cells, Monocytes, activated NK cells, activated Dendritic cells, resting Mast cells, activated Mast cells, and M2 macrophages were relatively lower (Figure 2F).

These findings underscore the pivotal role of altered immune cell composition in the progression of atheroma plaques, suggesting that immune infiltration may serve as a key driver of atherogenesis and a potential target for therapeutic intervention.

### WGCNA and identification of critical modules

**Figure 3:**
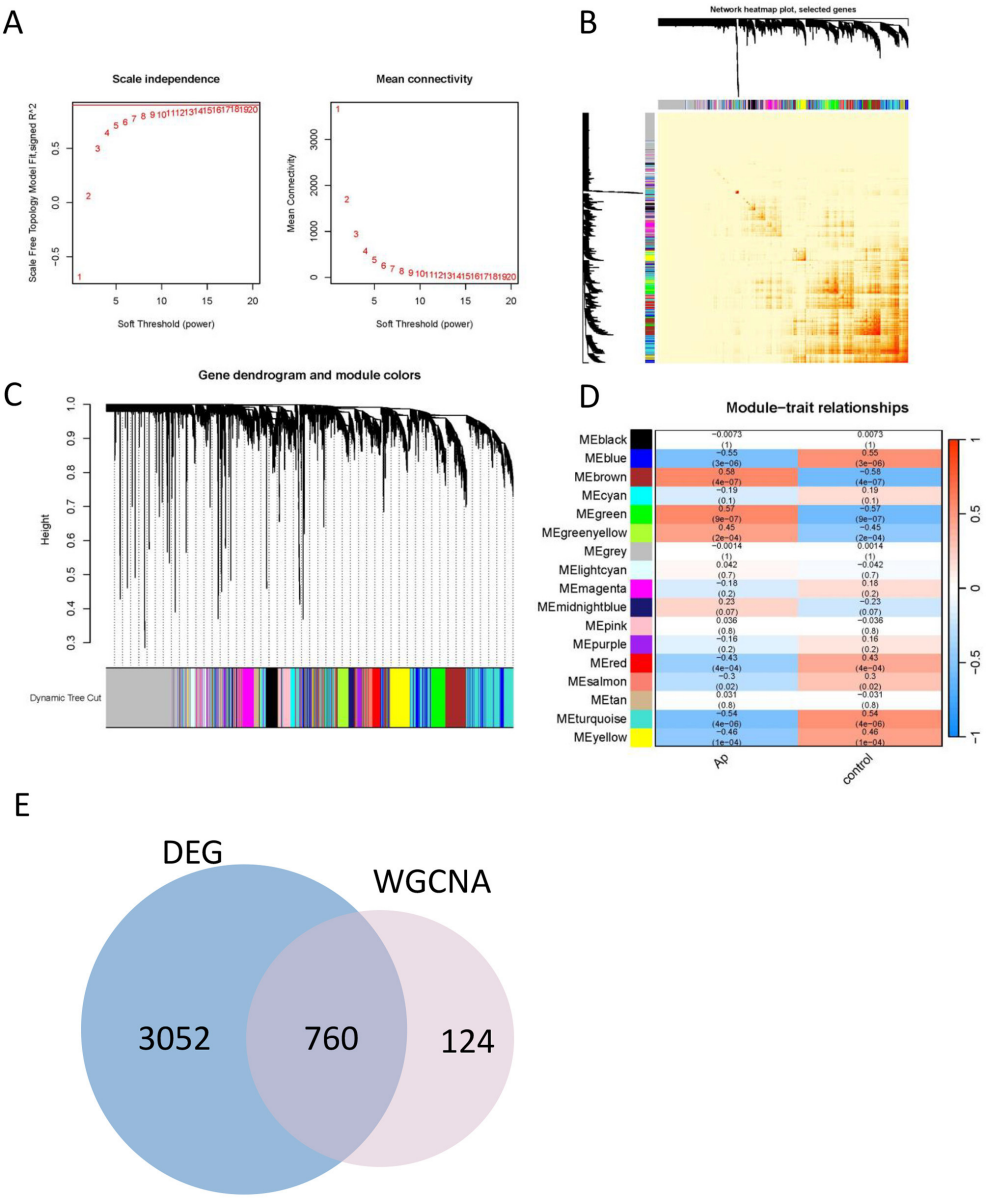
Construction of the co-expression network. **(A)** Soft threshold (power = 20) and scale-free topology fit index (*R*^2^ = 0.9). **(B)** The correlation heatmap of different modules. **(C)** Gene hierarchy tree clustering diagram. The graph indicates different genes horizontally and the uncorrelatedness between genes vertically, the lower the branch, the less uncorrelated the genes within the branch, i.e., the stronger the correlation. **(D)** Heatmap showing the relations between the modules and Ap feature. The value in the small cells of the graph represent the two-calculated correlation values cor coefficients between the eigenvalues of each trait and each module as well as the corresponding statistically significant *p*-values. Color corresponds to the size of the correlation; the darker the red, the more positive the correlation; the darker the green, the more negative the correlation. **(E)** Venn diagram for identification the overlapping genes from DEGs and WGCNA modules.

Weighted Gene Co-expression Network Analysis (WGCNA) was employed to construct a co-expression network based on the top 50% of genes with the highest variation in expression, that show substantial variation across the dataset, as they are more likely to reflect biological processes relevant to disease. Data quality from the 64 samples (including 32 Atheroma plaque and 32 intact tissue samples) was confirmed through cluster analysis. To establish a scale-free network, the soft threshold power β was set to 18, ensuring an independence degree of 0.9, and mean connectivity close to 0 (Figure 3A).

Genes exhibiting similar expression patterns were clustered into 17 distinct co-expression modules. The reliability of these modules was validated using hierarchical clustering, heatmaps, and adjacency relationship analyses (Figures 3B, C). Notably, the eigengenes of the brown and green modules demonstrated significant positive correlation with atheroma plaque (cor = 0.58, *P* = 4 × 10^−7^ and cor = 0.57, *P* = 9 × 10^−7^, respectively; Figure 3D).

These findings suggest that the brown module is likely to play a critical role in the progression of atheroma plaques. Consequently, the brown module was further analyzed to identify hub genes, which may serve as potential biomarkers or therapeutic targets for atherogenesis.

### Identification of the hub genes most associated with Ap

To elucidate the regulatory role of DEGs in the development of Ap, an intersection of DEGs and genes from the brown module was performed, resulting in the identification of 760 signature genes (Figure 3E). To refine the selection of core genes, LASSO regression and random forest analyses were utilized.

The LASSO model was used to perform feature selection by penalizing the coefficients of less informative variables, effectively shrinking them to zero. To validate the LASSO model, we performed 10-fold cross-validation (CV) during the training process. In this approach, the dataset was randomly partitioned into 10 subsets, and the model was trained and validated on each of these subsets, with 1 fold reserved for validation at each iteration. The average performance across the folds was used to assess the model's stability and predictive power.

The LASSO model uses an L1 penalty to shrink irrelevant coefficients to zero, effectively reducing the model's complexity and helping to avoid overfitting. For the Random Forest model, we adjusted the number of trees and the maximum depth of each tree to prevent excessively deep trees that might overfit the data. We also ensured that a sufficiently large number of trees were included to capture the underlying signal.

**Figure 4:**
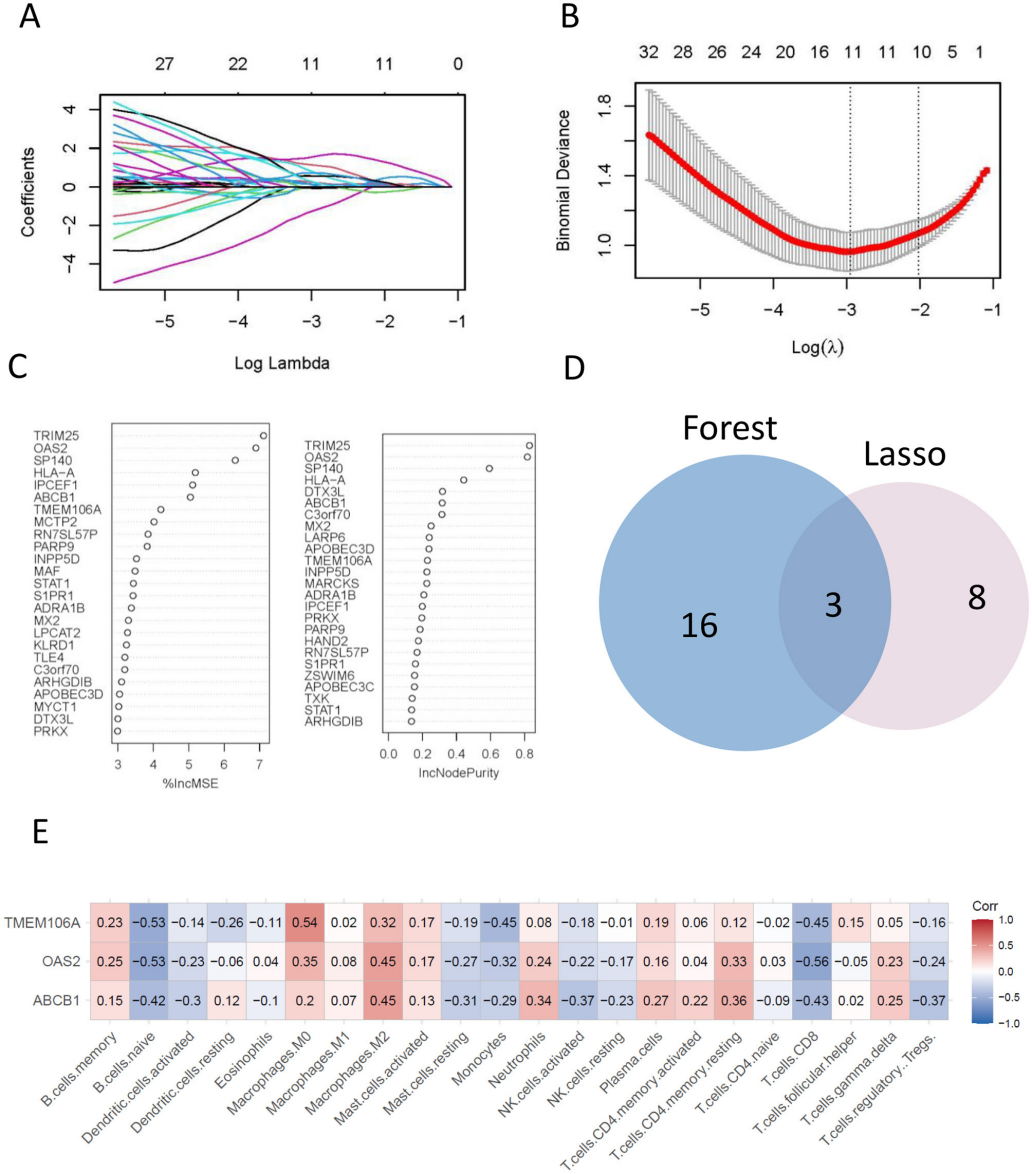
The final hub genes were identified by lasso regression and random forest analyses. **(A, B)** A least operator shrinkage and selection operator (LASSO) logistic regression was used to screen characteristic variables. **(C)** Ordination plot of gene importance scores. **(D)** Venn diagram showing the intersection feature variables filtered by the two algorithms. **(E)** The correlation between hub genes and the immune status was obtained by CIBERSORT algorithm analysis.

The LASSO regression algorithm identified 11 genes as potential biomarkers (Figures 4A, B), while the random forest (RF) algorithm highlighted 19 candidate genes (Figure 4C). By intersecting the results from the two methods (Figure 4D), three core genes were identified: OAS2, ABCB1, and TMEM106A.

These core genes represent key regulators associated with atheroma plaque development and may serve as promising biomarkers for diagnostic or therapeutic applications in atherosclerosis.

### Hub genes associations to immune cell infiltration

The associations between the expression levels of hub genes and the infiltration of immune cells were investigated using the CIBERSORT algorithm. The results revealed significant positive correlation between the expression levels of hub genes and the infiltration levels of immune cells, such as M0 macrophages, M2 macrophages, and resting memory CD4 T cells. Conversely, there was a notable negative correlation observed with the immune infiltration level of naive B cells, Monocytes, and CD8 T cells (Figure 4E). Notably, TMEM106A exhibited the highest positive correlation with M0 macrophages (Corr = 0.54) and a negative correlation with naive B cells (Corr = −0.53). Similarly, OAS2 showed a prominent positive correlation with M2 macrophages (Corr = 0.45) and a negative correlation with CD8 T cells (Corr = −0.56), while ABCB1 displayed a significant positive correlation with M2 macrophages (Corr = 0.45) and a negative correlation with CD8 T cells (Corr = −0.43).

These findings highlight the intricate relationship between hub gene expression and immune cell infiltration, suggesting that these genes may play critical roles in modulating the immune microenvironment of atheroma plaques. This underscores their potential as therapeutic targets for immune-based interventions in atherosclerosis.

### Identification the diagnostic ability of hub gene and the biology role of stroke

**Figure 5:**
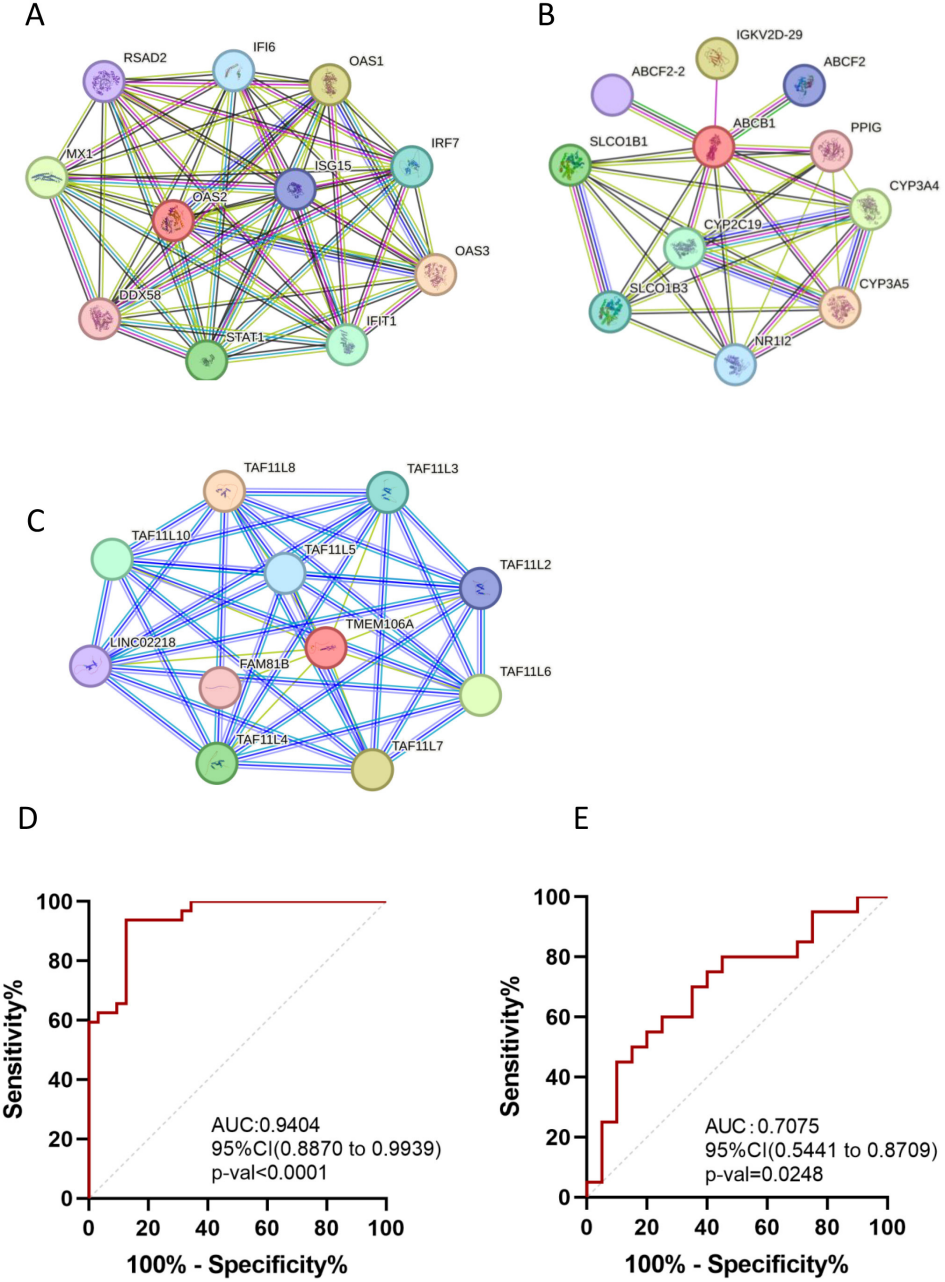
Protein-protein interaction network and diagnostic efficacy of OAS2. **(A–C)** protein-protein interaction network of OAS2, ABCB1, TMEM106A; **(D)** Receiver operating characteristic (ROC) curves assessing the diagnostic efficacy of OAS2, TMEM106A and ABCB1 in GSE43292 dataset. **(E)** Receiver operating characteristic (ROC) curves assessing the diagnostic efficacy of OAS2, TMEM106A and ABCB1 in GSE22255 dataset.

To investigate the biological functions of the identified hub genes in Ap and ischemic stroke, a protein-protein interaction (PPI) network was constructed using the STRING database with a medium confidence score threshold >0.4 (Figures 5A–C). The investigation revealed OAS2 involvement in the interleukin-27-mediated signaling pathway and the negative regulation of chemokine (C-X-C motif) ligand 2 production, highlighting its role in innate immunity responses. TMEM106A was shown to stimulate macrophages and drive them toward an M1-like phenotype via the activation of the MAPK and NF-kappaB signaling pathways.

These findings suggest that OAS2 and TMEM106A are involved in key immune regulatory processes that may contribute to the progression of atherosclerosis and ischemic stroke.

### Diagnostic potential of hub genes

The diagnostic capabilities of the identified hub genes (OAS2, TMEM106A, and ABCB1) were evaluated using a gene panel and receiver operating characteristic (ROC) curve analysis. For atherosclerotic plaques, the area under the curve (AUC) of 0.9404 (*p* < 0.0001; 95% CI: 0.887–0.9939), indicating excellent diagnostic potential (Figure 5D). For ischemic stroke, the AUC was calculated was 0.7075 (*p* = 0.0248; 95% CI: 0.5441–0.8709), suggesting moderate diagnostic and evaluative capabilities (Figure 5E).

**Figure 6:**
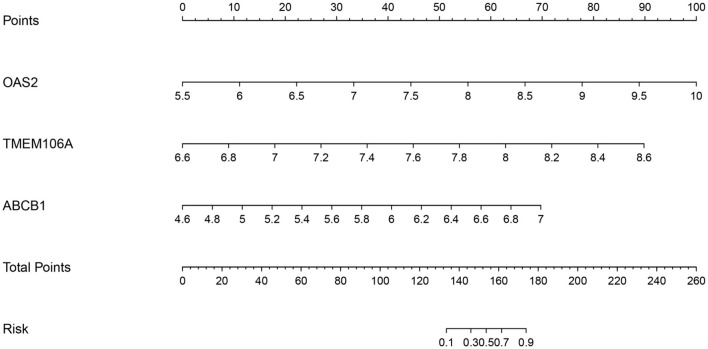
Nomogram for predicting the risk of ischemic stroke based on key gene expression.

To further assess ischemic stroke risk, a nomogram was constructed based on the expression levels of key genes OAS2, TMEM106A, and ABCB1. Gene-specific scores were calculated and summed to produce a total score reflecting an individual's stroke risk. This nomogram provides a practical tool for clinical risk assessment, with results visualized for ease of application (Figure 6).

These findings highlight the diagnostic value of the hub gene panel in both atherosclerosis and ischemic stroke, while also emphasizing the need for further research to elucidate the underlying mechanisms.

## Discussion

Ischemic stroke is a major global health issue that is intricately linked to the presence of atherosclerotic plaques. The accumulation of lipids and immune cells within these plaques significantly contributes to the pathogenesis of ischemic stroke by promoting inflammation and vascular instability. The identification of these connections underscores the potential for targeted therapeutic strategies aimed at both atherosclerosis and ischemic stroke prevention, paving the way for more effective management of these interconnected conditions.

In our investigation, we aimed to pinpoint hub genes associated with ischemic stroke derived from atheroma plaque and blood samples by employing a combination of WGCNA, the CIBERSORT method, and machine learning methodologies. We identified overlapping genes from the LASSO and random forest analyses as potential diagnostic markers, which included OAS2, TMEM106A, and ABCB1. However, the identification of hub genes represents only an initial step in understanding their roles in ischemic stroke. Further research is needed to establish their causal relationships with ischemic stroke and their potential as therapeutic targets.

The CIBERSORT algorithm was employed to analyze immune cell infiltration in individuals with atherosclerosis, revealing a marked distinction in immune cell infiltration patterns between atheroma plaque and intact tissue from atherosclerosis patients. Specifically, there was a notable contrast in the relative abundance of various cell types, including naive B cells, memory B cells, plasma cells, CD8 T cells, activated memory CD4 T cells, and M0 macrophages (Figure 2F). Subsequently, we conducted an immune infiltration analysis to investigate the relationship between OAS2, TMEM106A, and ABCB1 with immune cells. The findings indicate a significant correlation between hub genes and macrophages, CD8 T cells, and monocytes (Figure 4E).

Determining whether these genes have a causative link with ischemic stroke is essential for evaluating their potential as therapeutic targets. Recent studies and information from UniProt indicate that TMEM106A can stimulate macrophages and promote their differentiation into an M1-like phenotype via the activation of the MAPK and NF-kappaB signaling pathways (28, 29). This activation leads to the release of pro-inflammatory cytokines, including TNF, IL1B, IL6, CCL2 and nitric oxide (30, 31). Given that atherosclerosis is a chronic inflammatory condition, and that atheroma plaques significantly contribute to ischemic stroke, we propose that TMEM106A plays a crucial role in the progression of ischemic stroke.

Our study suggests that OAS2 is involved in the signaling pathway mediated by interleukin-27, leading to the downregulation of chemokine (C-X-C motif) ligand 2 production. Both of these factors play crucial roles in immune responses. Evidence indicates that OAS2 can be stimulated not only by IFNβ, but also by psoriasis-associated cytokines such as IL-17A and IL-6 (32, 33). Research conducted by Zhou et al. indicates that OAS2 may serve as a predictor for the severity and activity of psoriasis, and it could also be utilized as a marker to assess or monitor the effectiveness of clinical treatments (34). Considering that the immune response is a pivotal mechanism in ischemic stroke, it can be inferred that OAS2 has a significant impact on the progression of this condition.

Furthermore, we established a gene panel that includes OAS2, TMEM106A, and ABCB1 to evaluate their potential in predicting the diagnosis of atherosclerotic plaques and ischemic stroke. The calculated AUC was 0.9404, indicating that this gene panel exhibits promising diagnostic and evaluative capabilities for atherosclerotic plaques. In parallel, with an AUC of 0.7075, the gene panel also demonstrates notable diagnostic potential for ischemic stroke. Given the complex interplay between circulating blood and localized lesions, as well as the challenge in accurately reflecting local lesion conditions using blood markers, it is not surprising that the diagnostic performance of the gene panel in the blood of stroke patients shows a decline.

Recent studies, such as those by Zhang et al. (35) and Wang et al. (5), have successfully applied bioinformatics and machine learning to identify biomarkers for atherosclerosis or ischemic stroke. These studies focused on biomarkers for either atherosclerosis or ischemic stroke. Our work is novel in identifying shared diagnostic markers (OAS2, TMEM106A, ABCB1) that bridge both conditions, reflecting their clinical comorbidity and overlapping inflammatory pathways. Our machine learning model achieved an AUC of 0.9404 for diagnosing atherosclerotic plaques, outperforming previous studies. This improvement stems from our hybrid approach, which prioritizes genes with both high connectivity in co-expression networks and strong correlations to immune infiltration. While earlier works emphasized well-known inflammatory markers (e.g., IL6, TNF-α), we identified novel candidates (OAS2, TMEM106A, ABCB1) with limited prior links to atherosclerosis or stroke. For example, TMEM106A has been studied in neurodegeneration but not in vascular diseases, highlighting the originality of our findings.

While our study has yielded promising findings, it is crucial to acknowledge its limitations. One key limitation is the relatively small sample size, which may have affected the statistical power of our analysis. Consequently, the results should be interpreted with caution. Despite these limitations, our study offers a valuable starting point for further exploration in this field. We remain dedicated to advancing our understanding of these complex diseases and working toward the development of more effective diagnostic and therapeutic strategies.

As we continue to deepen our understanding of the genetic underpinnings of atherosclerotic plaques and ischemic stroke, the potential for breakthroughs in diagnosis and treatment expands. Our study, which identifies OAS2, TMEM106A, and ABCB1 as pivotal players in these conditions, contributes to a growing body of knowledge that could fundamentally transform our approach to cardiovascular health.

## Conclusion

Our study identified key genes associated with ischemic stroke from atheroma plaque and blood samples by employing WGCNA and machine learning methods. By establishing a gene panel comprising OAS2, TMEM106A, and ABCB1 for diagnostic prediction, we have opened new avenues for early detection and intervention. These findings could prove instrumental in the developing novel therapeutic strategies, potentially transforming the management of these complex diseases. Furthermore, our results indicate that OAS2, TMEM106A, and ABCB1 may serve as promising candidates for the diagnosis and assessment of atherosclerotic plaques and ischemic stroke. Despite some limitations in our study, these discoveries provide new mechanisms and potential targets for the prevention and treatment of ischemic stroke.

## Data Availability

The original contributions presented in the study are included in the article/Supplementary material, further inquiries can be directed to the corresponding author.
